# Maternal Smoking Highly Affects the Function, Membrane Integrity, and Rheological Properties in Fetal Red Blood Cells

**DOI:** 10.1155/2019/1509798

**Published:** 2019-11-29

**Authors:** Krisztina N. Dugmonits, Payal Chakraborty, Réka Hollandi, Szabolcs Zahorán, Gabriella Pankotai-Bodó, Péter Horváth, Hajnalka Orvos, Edit Hermesz

**Affiliations:** ^1^Department of Biochemistry and Molecular Biology, Faculty of Science and Informatics, University of Szeged, H-6726 Szeged, Közép fasor 52, Hungary; ^2^Institute of Biochemistry, Biological Research Centre, Hungarian Academy of Sciences, Szeged, Hungary; ^3^Department of Pathology, Faculty of Medicine, University of Szeged, Szeged, Hungary; ^4^Department of Obstetrics and Gynecology, Faculty of Medicine, University of Szeged, Szeged, Hungary

## Abstract

An understanding of the basic pathophysiological mechanisms of neonatal diseases necessitates detailed knowledge about the wide range of complications in the circulating fetal RBCs. Recent publications on adult red blood cells (RBCs) provide evidence that RBCs carry an active nitric oxide synthase (NOS3) enzyme and contribute to vascular functioning and integrity via their active nitric oxide synthesis. The aim of this study was to determine the effect of maternal smoking on the phenotypical appearance and functionality of fetal RBCs, based on morphological and molecular studies. We looked for possible links between vascular dysfunction and NOS3 expression and activation and its regulation by arginase (ARG1). Significant morphological and functional differences were found between fetal RBCs isolated from the arterial cord blood of neonates born to nonsmoking (RBC-NS, *n* = 62) and heavy-smoking (RBC-S, *n* = 51) mothers. Morphological variations were quantified by Advanced Cell Classifier, microscopy-based intelligent analysis software. To investigate the relevance of the newly suggested “erythrocrine” function in fetal RBCs, we measured the levels of NOS3 and its phosphorylation in parallel with the level of ARG1, as one of the major influencers of NOS3 dimerization, by fluorescence-activated cell sorting. Fetal RBCs, even the “healthy-looking” biconcave-shaped type, exhibited impaired NOS3 activation in the RBC-S population, which was paralleled with elevated ARG1 level, thus suggesting an increased redox burden. Our molecular data indicate that maternal smoking can exert marked effects on the circulating fetal RBCs, which could have a consequence on the outcome of in utero development. We hypothesize that any endothelial dysfunction altering NO production/bioavailability can be sensed by circulating fetal RBCs. Hence, we are putting forward the idea that neonatal RBC could serve as a real-time sensor for not only monitoring RBC-linked anomalies but also predicting the overall status of the vascular microenvironment.

## 1. Introduction

There is increasing evidence that environmental agents can exert a marked impact on the outcome of *in utero* development and even mediate long-lasting health consequences [1]. Many of the compounds present in cigarette smoke are regarded as harmful toxicants playing crucial roles in the pathogenesis of certain severe illnesses [2]. It has been hypothesized that many of the adverse effects may result from oxidative damage to proteins, lipids, and nucleic acids. Such damages could be traced back to direct attack of oxidants present in cigarette smoke and to the activation of the endogenous sources of reactive oxygen species (ROS) [3, 4].

Around 20-30% of women continue to smoke during full-term pregnancy [5]. The harmful pollutants in the vapor and tar phases might diffuse into the placenta and pass down to the fetal circulatory system through the umbilical cord. With the exception of its most proximal segment, the human umbilical cord lacks innervation and thereby the main regulator of their vascular tone and blood flow is the nitric oxide (NO), a signaling molecule produced by the endothelial nitric oxide synthase (NOS3) from L-arginine [6, 7].

Chronic smoking jeopardizes proper endothelial functioning by decreasing the formation and increasing the degradation of NO, via generation of ROS or other strong oxidants like peroxynitrite anion (ONOO^−^) [8].

The crucial function of NOS3 is the bioactive NO production, which depends on its subcellular localization and on the active dimeric conformational state [6]. NOS3 is dynamically regulated by cofactors like BH4, modulating the coupling of electron to the dimeric form [9], the availability of the substrate L-arginine [10], diverse interacting proteins [11], and the phosphorylation at Ser1177 residue via the PI3-kinase/Akt kinase pathway [12].

RBCs play an important role in tissue oxygenation and regulating blood pressure by metabolizing large quantities of NO. Our understanding on RBCs' biological function has been expanded by new groundbreaking data. Recently published experimental evidences make it highly supposable that adult RBCs not only are passive regulators of the endothelium-derived NO level but also actively control systemic NO bioavailability by synthesizing, transporting, and releasing it [13, 14]. This new “erythrocrine function” helps to maintain the vascular tone and blood flow by releasing bioavailable NO synthetized by the posttranslationally modified RBC-NOS3.

The RBCs' high nanomechanical plasticity or deformability allows them to be dynamically adapted to the continuous changing flow conditions along the vascular system. The nanomechanical properties of RBCs are highly compromised by any imbalance in the redox homeostasis; thus, continuous formation of oxidants is one of the important indicators of various pathological processes [15].

Fetuses are more sensitive to the toxicity of many substances, simply because of their lower detoxification and antioxidant capacities, lower immunologic competence, and higher proliferation rate. However, the physiological and the underlying molecular effects of tobacco smoke in particular on embryonic/fetal development still remain poorly understood.

We studied the consequences of an inappropriate maternal lifestyle, viz., smoking during pregnancy, on RBCs collected from umbilical cord arteries at the moment of birth. We looked for a potential rescue mechanism/compensatory role of fetal RBCs, based on the newly described “erythrocrine function” in case of any endothelial dysfunction.

## 2. Materials and Methods

### 2.1. Human Samples

Conforming to the principles outlined in the Declaration of Helsinki and with a pregnant mother's informed consent, blood was taken from the umbilical cord artery of neonates born to nonsmoking mothers (NSn = 62) and mothers who continued smoking habit during pregnancy (at least 10 cigarettes per day) (Sn = 51), in the Department of Obstetrics and Gynecology at the University of Szeged, Hungary. The Ethics Committee of the Department of Obstetrics and Gynecology approved the study protocol (16/2014).

The blood samples were centrifuged at 200*g* for 10 min at 4°C, and the lower two-thirds of the RBC phase was collected [13]. The RBCs were washed twice with 2 volumes of isotonic saline solution at pH = 7.0. The purity of the RBC preparation was checked by immunostaining with an RBC-specific mouse anti-glycophorin A antibody. Purity of the samples was >95% for RBCs.

### 2.2. Immunofluorescence Staining, Fluorescence-Activated Cell Sorting (FACS), and Microscopic Analysis

RBCs were fixed and stained as described by Chakraborty et al. [16]. Briefly, cells were fixed in 4% (*w*/*v*) paraformaldehyde in 0.05 M phosphate buffer (PB, pH = 7.4) at 4°C for 60 min. After extensive washing with PB, they were permeabilized with 0.1% Triton X-100 for 20 min and incubated for 1 h in PB containing 1% bovine serum albumin and 10% normal goat serum to block nonspecific antibody binding. RBCs were immunolabelled with primary antibodies (single or double staining) at 4°C overnight. Incubations with the primary antibodies was followed by washing and incubation with goat anti-mouse Alexa®647- and/or goat anti-rabbit Alexa®488-conjugated secondary antibodies for 1 h at room temperature (for the antibody list, see supplementary material online, [Supplementary-material supplementary-material-1]).

After washing, RBCs were either processed for quantitative analysis (FACS, BD FACSCalibur™, BD Biosciences) [17] or were mounted in ImmunoHistoMount (Sigma-Aldrich, Saint Louis, Missouri, USA) and examined under a confocal laser-scanning microscope (Zeiss LSM 880, Axiocam 503 mono, 40x oil immersion objective, numeric aperture: 1.4; Carl Zeiss Microscopy GmbH, Germany). Eight-bit pictures were taken by using ZEN 2.1 (black) software (Carl Zeiss Microscopy GmbH 1997-2015) and analyzed by using ImageJ 1.51n and ZEN 2.1.

### 2.3. *Ex Vivo* Experiments

#### 2.3.1. Heavy Metal Treatment

Total blood of four independent individuals (NS = 4, S = 4) was subjected to Cd^2+^treatment in an *ex vivo* experiment. Samples were incubated with and without cadmium acetate (Cd(CH_3_COO)_2_ × 2H_2_O in 0.9% NaCl) solution at the final concentration of 0.5 ng/*μ*L, 5 ng/*μ*L, 10 ng/*μ*L, 20 ng/*μ*L, and 50 ng/*μ*L for Cd^2+^ [18]. The incubation time was 30 minutes at 37°C. After treatment, morphological changes of parallel samples with and without Cd^2+^ were compared on eosin-stained blood smears.

#### 2.3.2. *Candida* Infection

Total blood of NS = 19 and S = 9 individuals was incubated for 20 hours at 37°C in a CO_2_ thermostat (5%) with and without *Candida parapsilosis* (ATCC 22019). *Candida parapsilosis* was maintained on YPD plates (1% yeast extract, 2% bactopeptone, 2% glucose, and 2.5% agar) at 4°C. Prior to the experiments, it was grown overnight in liquid YPD medium (1% yeast extract, 2% bactopeptone, and 2% glucose) at 37°C in a shaker incubator. Cells were harvested by centrifugation, washed twice with PBS (pH 7.4), and counted in a Bürker chamber, and the cell number was adjusted to 10^9^/mL. The final concentration was 10^7^ fungi/mL total blood. After treatment, morphological changes of the parallel samples were compared on eosin-stained blood smears.

After treatment, morphological changes of the parallel samples were compared on eosin-stained blood smears.

Eosin-stained smears' image processing was done in the scientific calculation software MATLAB. All the images went through quality control as a preprocessing step in the initial phase of the analysis, which consisted of illumination correction using the MATLAB tool CIDRE [19] and exclusion of poor-quality ones (e.g., too noisy or out-of-focus images).

The intelligent analysis software Advanced Cell Classifier [20] (ACC, available on http://cellclassifier.org) was utilized to (automatically) identify and populate distinct phenotypes present in the data, then refine decision planes between them with advanced machine learning methods such as active learning and neural networks, with minimal user interaction requirement. Based on such a model trained, all cells were predicted as a given phenotype. In data analysis, the reports created in ACC provided insight into the distribution of each phenotype of interest compared to the control one, while their mere frequency was also given.

### 2.4. Determination of ONOO^−^ Level

To determine the ONOO^−^ levels, we followed the method of Chakraborty et al. [16]. Spectrophotometric measurements were performed at 302 nm, using a GENESYS 10S UV-Vis spectrophotometer (Thermo Fisher Scientific, Madison, WI, USA). The total RBC fraction was hemolysed by the addition of distilled water at a ratio of 1 : 9. The hemolysate of each samples was diluted in 1 M NaOH solution in a ratio of 1 : 250. The increase in absorbance was measured until it reached a stable equilibrium; then, samples were added into 100 mM PB (pH = 7.4) in the same ratio as the reference. On this neutral pH, the ONOO^−^ decomposed, and the decrease in absorbance was observed till the equilibrium point [21]. The ONOO^−^ concentration was calculated as the difference in the absorbances at the two distinct pH values, according to the Lambert-Beer law (*Ɛ*_ONOO_^−^ = 1670 M^−1^ cm^−1^). The final results were normalized with protein concentration (*μ*mol/mg protein) [22].

### 2.5. Statistical Analysis

All statistical analyses were calculated with one-way analysis of variance (ANOVA) (GraphPad Statistical Software version 4.0) using the Newman-Keuls multiple comparison test. Significant differences were accepted at ^∗^*p* < 0.05, ^∗∗^*p* < 0.001, ^∗∗∗^*p* < 0.001, and ^∗∗∗∗^*p* < 0.0001.

## 3. Results

### 3.1. Characteristic Parameters of the Neonatal Study Populations

Mother age below 18 years, gestational age less than 37 weeks, complications during delivery, previous infection, inflammatory conditions or disorders such as cardiovascular diseases, diabetes mellitus, and malformations and evidence of genetic disorders were considered exclusion factors during sample collection. Significant differences were found between the NS and S study groups in the major independent characteristic parameters, such as the head and chest circumference, APGAR score at 1 min, and birthweight. The head and chest circumference values in the S group were 6-6.5% lower than those of NS origin. The overall birthweight difference was about 16% between the two examined groups (Table 1). Major difference was found in the birthweight-based distribution at weight under 2500 g and above 3500 g. 55% of neonates born to nonsmoking mothers had birthweight above 3500 g, while this value was only 13% in the case of neonates of smoking mothers (Suppl. [Supplementary-material supplementary-material-1]).

### 3.2. Increased Level of Morphological Abnormalities in RBC-S Populations

**Figure 1:**
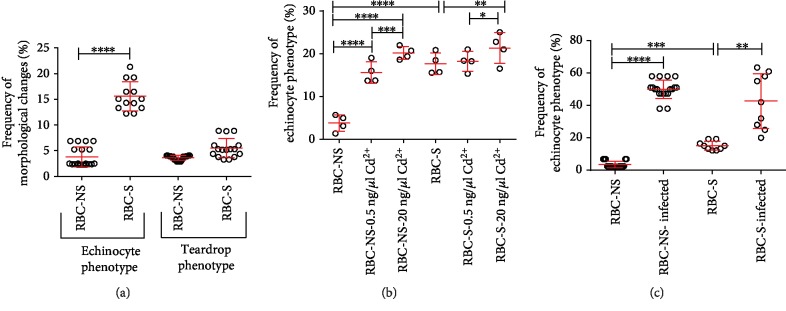
Distribution of phenotypic variations in RBC-NS and RBC-S populations. Frequencies of echinocyte and teardrop phenotypes in RBC-NS (*n* = 7956 cells from 19 individual samples) and RBC-S (*n* = 15745 cells from 45 individual samples) populations (a). Frequency of echinocytes (b), after incubation of total blood with Cd^2+^ at 0.5 and 20 ng/*μ*L concentration for 30 minutes at 37°C, RBC-NS (*n* = 7956 cells) and RBC-S (*n* = 15745 cells) investigated from 4 independent individuals in each group. *Candida parapsilosis* induced morphological changes in RBC-NS and RBC-S populations (c). Total blood samples were incubated for 20 hours at 37°C at 5% CO_2_ with and without *Candida parapsilosis* (ATCC 22019) (10^7^ fungi/1 mL). After the *ex vivo* treatment, morphological changes were counted in RBC-NS (*n* = 3548 cells from 19 independent samples) and RBC-S (*n* = 4173 cells from 9 independent samples) on eosin-stained blood smears. Phenotypic variants were analyzed by the Advanced Cell Classifier software. Statistical significances were accepted at ^∗^*p* < 0.05, ^∗∗^*p* < 0.01, ^∗∗∗^*p* < 0.001, and ^∗∗∗∗^*p* < 0.0001 by one-way ANOVA using the Newman-Keuls multiple comparison test.

Pathological conditions affecting RBCs can lead to significant alteration in their characteristic, biconcave disc shape, and deformability. Altogether, we screened over 25000 cells of RBC-NS (*n* = 7956 cells from 20 independent individuals) and RBC-S (*n* = 15745 cells from 17 independent individuals) origins for phenotypic variants, utilizing the Advanced Cell Classifier intelligent analysis software. In the RBC-S population, the echinocyte phenotype was about 15% of the total cell count, whereas in the RBC-NS group, the frequency of this phenotype was less than 2%. The frequency of teardrop-like RBCs showed an elevated tendency in the RBC-S population, but the increase was not significant (Figure 1(a)). To demonstrate that active cigarette smoking could be one of the etiological factors for the adverse outcomes in morphological changes, we induced controlled oxidative damage by incubating the total blood with and without Cd^2+^ in 0.5, 5, 10, 20, and 50 ng/*μ*L concentration for 30 minutes at 37°C in an *ex vivo* experiment. In the NS population, Cd^2+^, at a concentration as low as 0.5 ng/*μ*L, transformed 16-18% of RBCs to echinocytes. On the contrary, in the RBC-S population, we detected a modest increase (~5%) only at 20 ng/*μ*L Cd^2+^ concentration and above, compared to the appropriate control group without Cd^2+^ (Figure 1(b)). In parallel, we tested the effect on morphological appearance by an additional stressor, mimicking *Candida parapsilosis* infection, which was also reported to increase free radical production. *Candida* treatment induced morphological changes in both RBC populations, regardless of their origin, though cells in the NS population exhibited higher sensitivity (Figure 1(c)).

### 3.3. Decreased NOS3 Activation Indicates Impairment in the Functionality of Cells in the RBC-S Population

**Figure 2:**
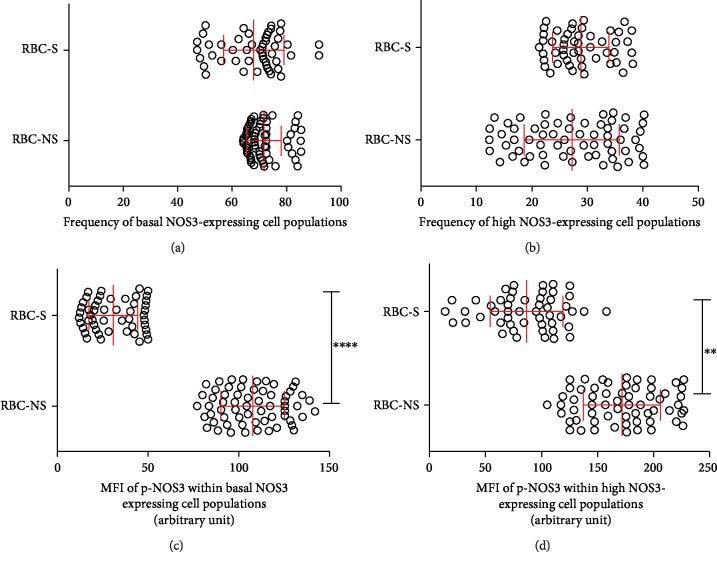
Quantification of the immunolabelled NOS3 from RBC-NS and RBC-S populations. Graphical representation of the frequency of basal (a) and high (b) NOS3-expressing cell populations in RBC-NS (*n* = 62 independent clinical subjects) and RBC-S (*n* = 51 independent clinical subjects), quantified by FACs analysis. Summary of NOS3 phosphorylation level (pNOS3) within basal (c) and high (d) NOS3-expressing cells. Statistical significances were accepted at ^∗∗^*p* < 0.01 and ^∗∗∗∗^*p* < 0.0001 by one-way ANOVA using the Newman-Keuls multiple comparison test. MFI: mean fluorescence intensity.

To investigate the relevance of the newly suggested “erythrocrine function” in fetal RBCs, we measured the NOS3 level and its phosphorylation status by FACS analysis. During the evaluation of the histograms, we set an intensity value of 10^2^ as a borderline between basal and high NOS3-expressing populations. Though the NOS3 expression in the RBC-S population was somewhat lower than that in the RBC-NS group, the difference was not statistically significant. Even the frequency of the basal and high NOS3-expressing cell was very similar between the two groups (2/3 and 1/3) (Figures 2(a) and 2(b) and Suppl. [Supplementary-material supplementary-material-1]). In parallel with NOS3 detection, we investigated the phosphorylation level at Ser1177. In general, NOS3 proteins were highly phosphorylated in the RBC-NS population, while in the RBC-S group, the phosphorylation lagged behind. Phosphorylation in the basal and high NOS3-expressing cells was about 25% and 50% of the levels detected in the matching RBC-NS populations, respectively (Figures 2(c) and 2(d) and Suppl. [Supplementary-material supplementary-material-1] and [Supplementary-material supplementary-material-1]).

**Figure 3:**
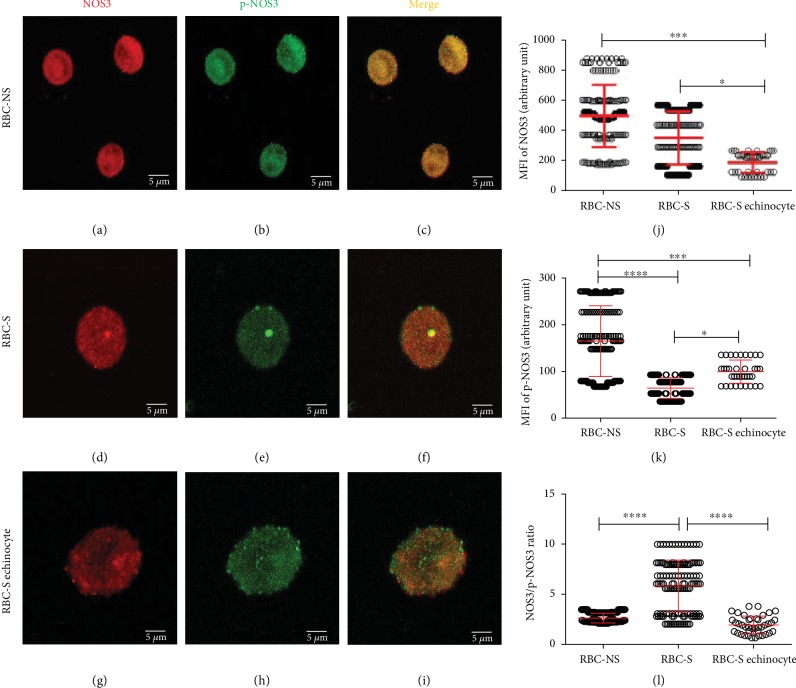
Visualization of the confocal images with varying morphology in relation to the phosphorylation of NOS3 in RBC-NS and RBC-S populations. The representative confocal *Z*-stack images show RBC-NS (a–c) and RBC-S (d–f) with regular biconcave and RBC-S echinocyte (g–i) with the echinocyte phenotypes. The panels (a, d, and g) show the RBCs immunolabelled with a mouse primary anti-NOS3 antibody followed by an Alexa Fluor® 647 secondary antibody. The panels (b, e, and h) represent the RBCs immunolabelled with a rabbit anti-p-NOS3 (p-NOS3) primary antibody, followed by an Alexa Fluor® 488 secondary antibody. The microscopic parameter, i.e., the thickness of *z*-axis, was 3.845 *μ*m/slice, and the scale bar of 5 *μ*m was kept constant at all instances. The RBCs were randomly selected from both RBC-NS (*n* = 40-45 regular biconcave-shaped cells from each of 3 independent samples) and RBC-S (*n* = 30-35 regular biconcave cells from each of 5 independent samples and *n* = 10 echinocytes from each of 4 independent samples) groups. In the highlighted zone of interest, zooming ratios were 4.486, 6.865, and 10.019 in (a)–(c), (d)–(f), and (g)–(i), respectively. The ImageJ 1.51n software was used to quantify the mean intensity levels from three middle slices in the RBC-NS, RBC-S, and RBC-S echinocyte for NOS3 (j) and p-NOS3 (k). (l**)** The ratio between NOS3 and p-NOS3 intensity levels. All statistical analyses were accepted by one-way ANOVA using the Newman-Keuls multiple comparison test at ^∗^*p* < 0.05, ^∗∗∗^*p* < 0.001, and ^∗∗∗∗^*p* < 0.0001. MFI: mean fluorescence intensity.

The shape of RBCs helps us to critically predict their biological function. Here, we looked for possible correlation between morphological variation and functional behavior of RBCs. In parallel with FACS analysis, anti-NOS3/p-NOS3 double-labelled RBCs were subjected to ImageJ evaluation of *Z*-stack confocal images (Figures 3(a)–3(i)). The phenotypic variants in the RBC-S population are affected at different levels causing impairment in NOS3 regulation. In the biconcave disc-shaped cells of RBC-S origin, there was no significant alteration detected in the NOS3 level, while the phosphorylation was about 50% of the morphologically matched RBC-NS cells (Figures 3(j) and 3(k)). The echinocytes of S origin exhibited low NOS3 expression, about 55% of the biconcave disc-shaped cells from either of origins (Figure 3(j)). This lowered NOS3 level was paralleled with lower phosphorylation (Figure 3(k)), and as a consequence, the NOS3/p-NOS3 ratio was comparable to that found in the biconcave disc-shaped cells with NS origin (Figure 3(l)).

### 3.4. Increased ARG1 Level Indicates an Altered NOS3 Activation

**Figure 4:**
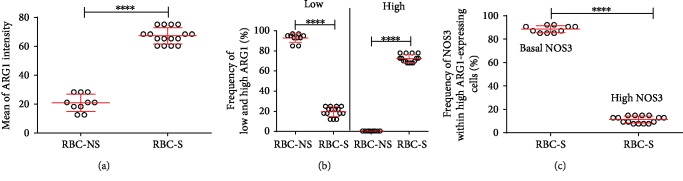
Quantification of the immunolabelled ARG1 cells by FACS analysis from RBC-NS- and RBC-S-derived samples. Graphical representation of the intensity (a) and frequency (b) of ARG1 expression level based on FACS analysis of cell populations, originated from RBC-NS (*n* = 10 independent clinical subjects) and RBC-S (*n* = 15 independent clinical subjects) samples. (c) The NOS3 intensity level within the high ARG1-expressing RBC-S population. Statistical significances were accepted at ^∗∗∗∗^*p* < 0.0001 by one-way ANOVA using the Newman-Keuls multiple comparison test.

Adult human RBCs carry a functional ARG1 enzyme, which competes with NOS3 for their common substrate, L-arginine. We also could detect ARG1 in fetal RBCs. To assess the alterations that may underlie the difference in NOS3 activation between groups RBC-NS and RBC-S, we followed ARG1 and NOS3 coexpression, measuring the frequencies and intensities of ARG1- and NOS3-positive RBCs by FACS analysis. The ARG1 expression was generally low in the RBC-NS group; 98% of the cells can be characterized with low ARG1 level (Figures 4(a) and 4(b)). RBCs with S origin demonstrated a significantly higher ARG1 expression. In about 75% of the cells, we detected high ARG1 level (Figure 4(b)), and around 90% of these high ARG1-expressing cells expressed the basal level of NOS3 (Figure 4(c) and Suppl. [Supplementary-material supplementary-material-1]).

### 3.5. Elevated ONOO^−^ Level Indicates an Increase in Free Radical Production

**Figure 5:**
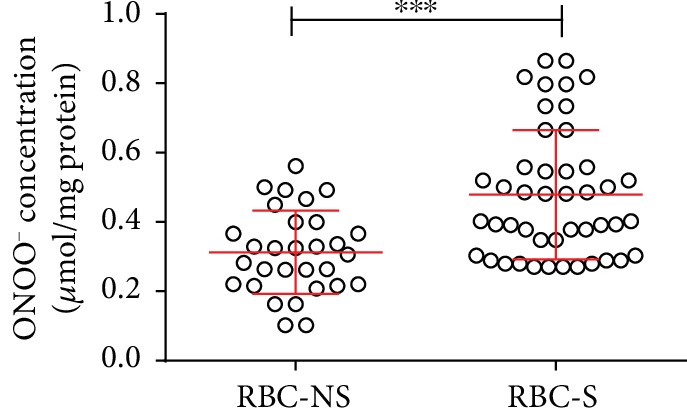
Spectrophotometric measurements of the strong oxidant in RBC-NS and RBC-S populations. Peroxynitrite (ONOO^−^) levels were measured in RBCs from the umbilical cord arteries of independent clinical subjects in each group with NS and S origin (*n* = 10 for RBC-NS and *n* = 15 for RBC-S). Statistical significance was accepted at ^∗∗∗^*p* < 0.001 by one-way ANOVA using the Newman-Keuls multiple comparison test.

In case of impaired activation, NOS3 is unable to produce bioavailable NO but increases the production of superoxide radicals leading to the imbalance of the redox homeostasis. Cells of RBC-S origin had a significantly increased level of ONOO^−^ (~1.5-fold) (Figure 5).

## 4. Discussion

Sustained maternal smoking had been escalated to a worldwide health issue, with insufficient oxygen supply that can induce marked effects on the *in utero* development [5, 23, 24]. Smoking-induced vascular endothelial dysfunction highlights the circulating RBCs as vital sensor entities, in the purview to operate any compensatory mechanism [14, 25, 26]. This project was based on the expectation that the status of the circulating fetal RBCs will be reflecting the consequences on a long-term exposure to the harmful substances that originated from an improper maternal lifestyle and remained unfiltered by the placental barrier.

Our results indicated that RBCs of the developing fetus born to a smoking mother undergo morphological and molecular alterations/aberration. We demonstrated that fetal RBCs carry functional NOS3, and during long-term *in vivo* exposure to harmful stimuli, the NOS3 level and its activation pathways are altered in a morphology-dependent manner. Moreover, ARG1 level is elevated in fetal RBCs of smoking origin and high ARG1-expressing cells show low NOS3 level. We also demonstrated that cells in the RBC-S population become a source of oxidizing agents, carrying the possibility of further inactivation of the NOS3 pathway by induced ARG1 level.

The phenotypic variations in the RBC-S population, even the characteristic biconcave disc-shaped cells, can be characterized with impaired NOS3 activation. One of the critical influential steps in NOS3 activation is the phosphorylation of the Ser1177 residue via phosphatidylinositol-3 kinase and the downstream serine/threonine protein kinase Akt pathway [12]. In the RBC-S populations, the bulk of the healthy-looking biconcave disc-shaped cells showed a significantly lower level of phosphorylation, compared to their morphological match in the control population. Contrarily, in the increased echinocyte population, probably the phosphorylation pathway was unaltered but the NOS3 level was significantly lowered. This kind of alteration could be explained by either an altered *de novo* biosynthesis or an enhanced rate of NOS3 monomerization, accompanied by NOS3 degradation [27].

In clinical practice, phenotypic variants of RBCs serve as a preliminary platform for many clinical diagnoses [28–32]. However, recognizing and understanding the molecular alterations in RBCs preceding the detectable phenotypic changes would allow us to recognize and intervene into the possible complications at earlier stages. In this study, we provided evidence that RBCs could be functionally impaired before their detectable morphological alterations. Phenotypical variants are connected to changes in the cytoskeletal network. We have no supporting data about the timing and extent of these changes, which marks as the limitation to our conclusion. However, the changes in NOS3/p-NOS3 could serve as an early marker alerting for possible risk in later development.

Additional evidence also supports our conclusion that before the appearance of obvious phenotypic variants, RBCs are already influenced in their functional capacity by maternal smoking. The *ex vivo* treatment of RBCs with the oxidizing agent cadmium indicated that cells in the RBC-S population developed resistance against metal or metal-induced oxidative insults, regardless of the phenotypic appearance. There were almost two orders of magnitude difference in Cd^2+^ concentration necessary to induce the echinocyte phenotype between the NS and S populations. On the other hand, we did not observe significant protection against the fungal infection, with known harmful outcome on RBC's membrane [33]; it led to an increased number of echinocytes in both the NS and S populations.

Another important regulator molecule in the NOS3-NO production pathway is the ARG1 enzyme. It metabolizes L-arginine to urea and L-ornithine. Enhanced ARG level or activity has been implicated in many diseases including cardiovascular dysfunction [34–36], partly because the excessive production of ornithine may be involved in the formation of vascular structural problems [37]. On the other hand, the competition between NOS3 and ARG1 for their common substrate L-arginine could lead to the impairment of NO production [38], because the maximal catalytic rate of ARG1 is at least 1000-fold of NOS3 [39]. Recent studies provided novel insights into oxidative stress increasing ARG1 expression in endothelial cells [40–42], and the upregulation of ARG impairs endothelium-dependent NO-mediated dilatation [40]. Continuously growing data indicate that RBCs themselves are able to contribute to vascular functioning and integrity; a NOS3-derived NO export from RBCs mediates protection against vascular injury. This protective effect of RBC-NOS3 can be tightly regulated by ARG1 in an acute oxidative stress condition with excessive overproduction of ROS [25, 43]. Under a pathological condition, an intimate crosstalk between dysfunctional endothelial cells and the circulating RBCs was recently reported where RBCs originated from patients with type 2 diabetes-induced vascular endothelial dysfunction in healthy rat aorta via upregulated vascular ARG1. This induced dysfunctionality can be prevented by inhibition of ARG activity and/or increased ROS production [25].

We could detect ARG1 in fetal RBCs and demonstrated that ARG1 is upregulated in the RBC-S populations. It is well known that pregnancy itself is associated with an enhanced metabolism and demand for O_2_, which may lead to the overproduction of ROS and the development of oxidative stress. Maternal smoking during pregnancy leads to an extra source of ROS because the unfiltered prooxidants/oxidants could be a direct inducer of ARG1 in the RBC-S population. Another possibility for ARG upregulation is an elevated intrinsic ROS source from reduction of BH4 cofactor to BH2. Under this condition, NOS3 uncoupling may occur, resulting in the formation of superoxide anion (O_2_^·-^) instead of NO [10]. The O_2_^·-^ itself and the highly cytotoxic oxidant ONOO^−^, formed in a spontaneous reaction of O_2_^·-^ with NO [8] are the potential inducers of ARG1 [40].

Recent publications already demonstrated in the adult system that ARG plays an essential role in the regulation of the complex mechanism of NOS3 uncoupling under various pathological conditions [42] and that NOS3 uncoupling forms a key step in the progression of major vascular dysfunction [44]. Cortese-Krott and Kelm published decreased RBC-NOS3 expression and activity in correlation with endothelial dysfunction in case of coronary artery disease [14]. Based on our data, it is most likely that fetal RBCs also lose their NOS3 activity under certain pathological circumstances.

## 5. Conclusion

**Figure 6:**
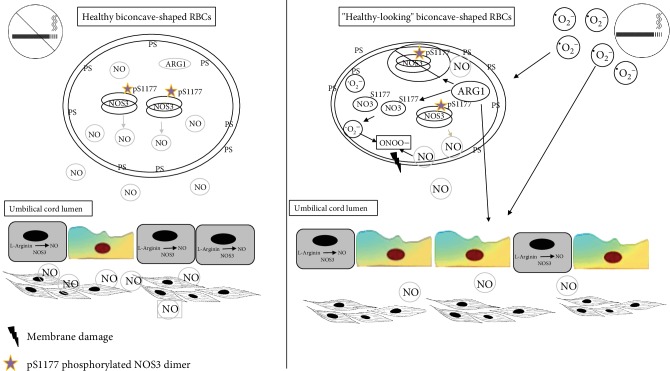
Graphical summary.

In this work, we demonstrated that RBCs from the smoker origin become a source of reactive oxygen and nitrogen species and lose their characteristic structural and functional features. Assuming an intimate crosstalk between the vascular endothelium and the circulating RBCs, any endothelial dysfunction altering NO production/bioavailability can be sensed by fetal RBCs acting as a biosensor; however, in the RBC-S population, the NOS3-NO production is most likely unavailable as a compensatory mechanism to improve the blood flow to the fetus. Moreover, the changed protein expression profile might even augment and synergizes the prognosis of vascular dysfunction/comorbidities (for a graphical summary, see Figure 6).

## Figures and Tables

**Table 1:** Parameters of the study groups.

	Neonates born to nonsmoking mothers	Neonates born to smoking mothers
*n*	62	51
Birthweight (kg)	3.4883 ± 0.4465	2.9469±0.5850^∗∗∗∗^
Gestational age at delivery (week)	39.295 ± 1.252	38.492 ± 2.481
Blood sample pH	7.277 ± 0.068	7.283 ± 0.080
Chest circumference (cm)	33.083 ± 1.763	31.101±2.802^∗∗∗∗^
Head circumference (cm)	34.65 ± 1.400	32.575±1.873^∗∗∗∗^
APGAR score at 1 min	9.33 ± 0.90	8.969 ± 1.189^∗^
Maternal age (years)	32.453 ± 5.637	28.015 ± 5.729

Total blood samples were collected from the umbilical cord arteries of neonates born to nonsmoking (NS, *n* = 62) and smoking (S, *n* = 51) mothers and analyzed according to the experimental protocols. The data are expressed as means ± standard deviation. Statistical significances were accepted at ^∗^*p* < 0.05 and ^∗∗∗∗^*p* < 0.0001 by one-way ANOVA using the Newman-Keuls multiple comparison test. APGAR: appearance, pulse, grimace, activity, and respiration.

## Data Availability

The data used to support the findings of this study are available from the corresponding author upon request.

## Supplementary Materials

Supplementary Materials Table 1. List of the sources and dilutions of primary and secondary antibodies. Figure S1. Birthweight-based distribution of neonates with nonsmoking and smoking origins. Figure S2. Quantification of the immunolabelled NOS3 and p-NOS3 by FACS analysis from RBC-NS and RBC-S populations. Figure S3. Quantification of the immunolabelled ARG1 by FACS analysis from RBC-NS- and RBC-S-derived samples.
