# Erratum to “Maternal Smoking Highly Affects the Function, Membrane Integrity, and Rheological Properties in Fetal Red Blood Cells”

**DOI:** 10.1155/2021/9762481

**Published:** 2021-03-25

**Authors:** Krisztina N. Dugmonits, Payal Chakraborty, Réka Hollandi, Szabolcs Zahorán, Gabriella Pankotai-Bodó, Péter Horváth, Hajnalka Orvos, Edit Hermesz

**Affiliations:** ^1^Department of Biochemistry and Molecular Biology, Faculty of Science and Informatics, University of Szeged, H-6726 Szeged, Közép fasor 52, Hungary; ^2^Institute of Biochemistry, Biological Research Centre, Hungarian Academy of Sciences, Szeged, Hungary; ^3^Department of Pathology, Faculty of Medicine, University of Szeged, Szeged, Hungary; ^4^Department of Obstetrics and Gynecology, Faculty of Medicine, University of Szeged, Szeged, Hungary

In the article titled “Maternal Smoking Highly Affects the Function, Membrane Integrity, and Rheological Properties in Fetal Red Blood Cells” [1], there was an error in Figure 3. The figure should show a single cell image with 5 *μ*m scale bar. The error was introduced during the production process of the article, and Hindawi apologises for this. The corrected figure is shown below and is listed as Figure 1:

## Figures and Tables

**Figure 1:**
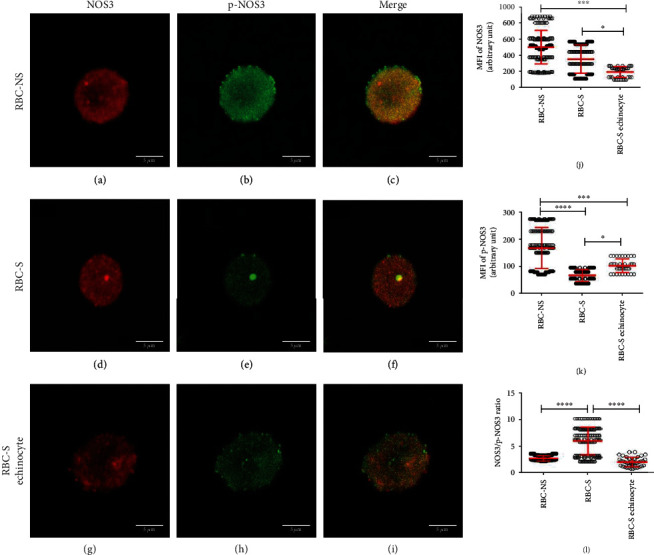
Visualization of the confocal images with varying morphology in relation to the phosphorylation of NOS3 in RBC-NS and RBC-S populations. The representative confocal Z-stack images show RBC-NS (a–c) and RBC-S (d–f) with regular biconcave and RBC-S echinocyte (g–i) with the echinocyte phenotypes. (a, d, g) show the RBCs immunolabelled with a mouse primary anti-NOS3 antibody followed by an Alexa Fluor® 647 secondary antibody. (b, e, h) represent the RBCs immunolabelled with a rabbit anti-p-NOS3 (p-NOS3) primary antibody, followed by an Alexa Fluor® 488 secondary antibody. The microscopic parameter, i.e., the thickness of *z*-axis, was 3.845 *μ*m/slice, and the scale bar of 5 *μ*m was kept constant at all instances. The RBCs were randomly selected from both RBCNS (*n* = 40‐45 regular biconcave-shaped cells from each of 3 independent samples) and RBC-S (*n* = 30‐35 regular biconcave cells from each of 5 independent samples and *n* = 10 echinocytes from each of 4 independent samples) groups. In the highlighted zone of interest, zooming ratios were 4.486, 6.865, and 10.019 in (a–c), (d–f), and (g–i), respectively. The ImageJ 1.51n software was used to quantify the mean intensity levels from three middle slices in the RBC-NS, RBC-S, and RBC-S echinocyte for NOS3 (j) and p-NOS3 (k). (l) The ratio between NOS3 and p-NOS3 intensity levels. All statistical analyses were accepted by one-way ANOVA using the Newman-Keuls multiple comparison test at ^∗^*p* < 0.05, ^∗∗∗^*p* < 0.001, and ^∗∗∗∗^*p* < 0.0001. MFI: mean fluorescence intensity.
