# Supplementary material for: Maternal Smoking Highly Affects the Function, Membrane Integrity, and Rheological Properties in Fetal Red Blood Cells
Source: Oxid Med Cell Longev. 2019 Nov 29;2019:1509798. doi: 10.1155/2019/1509798 (PMC6906794; doi:10.1155/2019/1509798)
Supplement: Supplementary Materials — Table 1. List of the sources and dilutions of primary and secondary antibodies. Figure S1. Birthweight-based distribution of neonates with nonsmoking and smoking origins. Figure S2. Quantification of the immunolabelled NOS3 and p-NOS3 by FACS analysis from RBC-NS and RBC-S populations. Figure S3. Quantification of the immunolabelled ARG1 by FACS analysis from RBC-NS- and RBC-S-derived samples. [file 1509798.f1.docx]

**Supplementary Materials**

Maternal smoking highly affects the function, membrane integrity and rheological properties in fetal red blood cells

Krisztina N. Dugmonits, Payal Chakraborty, Réka Hollandi, Szabolcs Zahorán, Gabriella Pankotai-Bodó, Péter Horváth, Hajnalka Orvos, Edit Hermesz

Table of Contents:

[TABLE 1: List of the sources and dilutions of primary and secondary antibodies. 2](#_Toc19087448)

[FIGURE S1: Frequency of neonates’ birthweight with non-smoking and smoking origin 2](#_Toc19087449)

[FIGURE S2: Quantification of the immunolabelled NOS3 and p-NOS3 by FACS analysis from RBC-NS and RBC-S populations 3](#_Toc19087450)

[FIGURE S3: Quantification of the immunolabelled ARG-1 by FACS analysis from RBC-NS and RBC-S derived samples 3](#_Toc19087451)

| **Antibody** | **Host** | **Dilution** | **Code** | **Distributor** |
| --- | --- | --- | --- | --- |
| anti-Glycophorin A | mouse | 1:50 | MA5-12484 | Thermo Fisher Scientific, Madison, WI, USA |
| anti-NOS3 | mouse | 1:200 | sc-376751 | Santa Cruz Biotechnology Inc., Dallas, TX, USA |
| anti-NOS3 | rabbit | 1:100 | ab5589 | Abcam, Cambridge, UK |
| anti-pSer1177 NOS3 | rabbit | 1:100 | SAB-4300128 | Sigma Aldrich, Saint Louis, Missouri, USA |
| anti-Arginase-1 | rabbit | 1:100 | AVI 3058 G | Biocare Medical, Pacheco, CA, USA |
| anti 4-hydroxy-2-nonenal | mouse | 1:100 | ab48506 | Abcam, Cambridge, UK |
| goat anti-mouse Alexa®647 | mouse | 1:400 | ab150115 | Abcam, Cambridge, UK |
| goat anti-rabbit Alexa®488 | rabbit | 1:400 | ab150077 | Abcam, Cambridge, UK |

# TABLE 1: List of the sources and dilutions of primary and secondary antibodies.

**
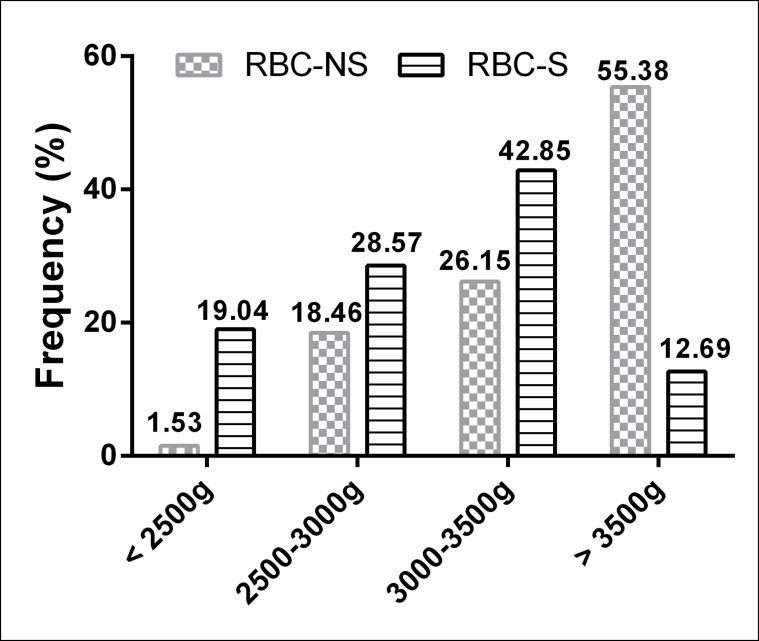
**

#

# FIGURE S1: Frequency of neonates’ birthweight with non-smoking and smoking origin

Samples with non-smoking (n=62 independent clinical volunteers) and smoking origin (n= 51 independent clinical volunteers) were divided into 4 groups based on their birthweight. Values above columns indicate the percentage of neonates in the given weight-range. In all the categories there was significant difference between the neonates of non-smoking and smoking origin.


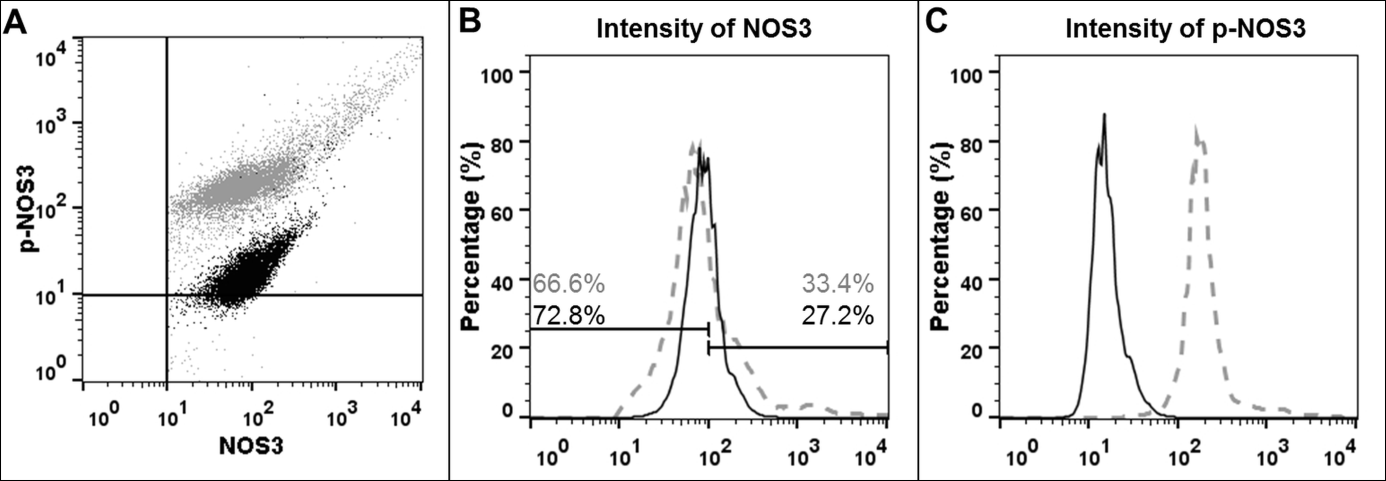


#

# FIGURE S2: Quantification of the immunolabelled NOS3 and p-NOS3 by FACS analysis from RBC-NS and RBC-S populations

Representative dot plot (**A**) and histograms (**B and C**) from FACS analysis using anti-NOS3 and anti-p-NOS3 primary antibodies on RBC-NS (grey dots) and RBC-S (black line) populations; 10^2^ value was arbitrarily applied as a borderline between basal and high NOS3 (**B**) and p-NOS3 (**C**) intensities.


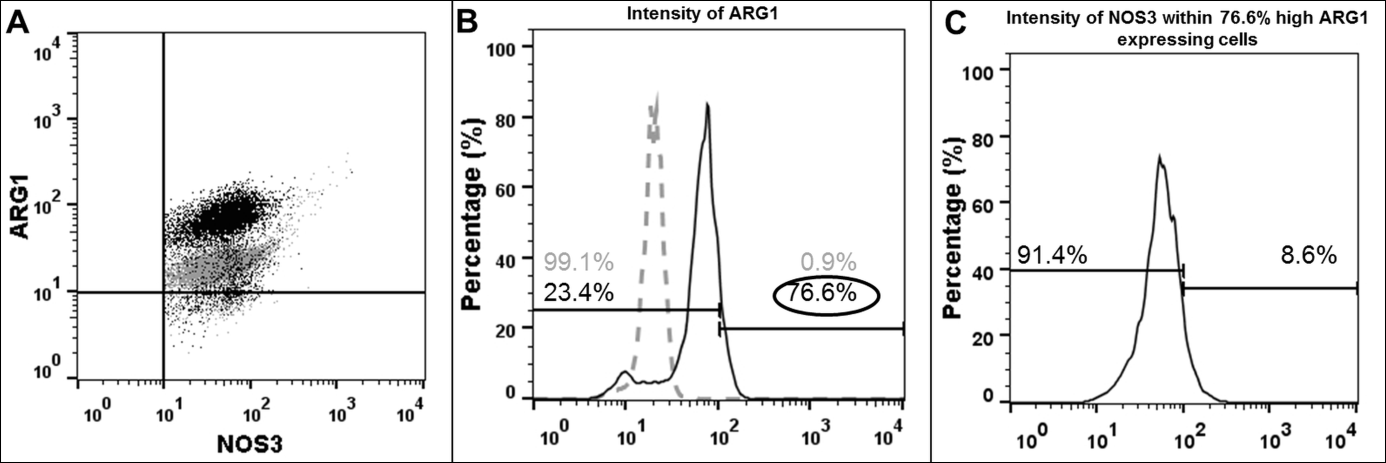


#

# FIGURE S3: Quantification of the immunolabelled ARG-1 by FACS analysis from RBC-NS and RBC-S derived samples

Representative dot plot (**A)** and histograms (**B** and **C**) from FACS analysis on ARG-1 and NOS3, originated from RBC-NS (grey dots) and RBC-S (black line) samples. Panel (**B**) shows the ARG-1 intensity level, on the x-axis 5x10^1^ value was arbitrarily applied as a borderline between basal and high level of ARG-1 intensity. (**C**) represent the NOS3 intensity level within the high ARG-1 expressing RBC-S population, where the values below and above 10^2^ denote the frequencies of basal and high NOS3 expressing cell population, respectively.
